# GZD824 suppresses the growth of human B cell precursor acute lymphoblastic leukemia cells by inhibiting the SRC kinase and PI3K/AKT pathways

**DOI:** 10.18632/oncotarget.10881

**Published:** 2016-07-28

**Authors:** Wei Ye, Zhiwu Jiang, Xiaoyun Lu, Xiaomei Ren, Manman Deng, Shouheng Lin, Yiren Xiao, Simiao Lin, Suna Wang, Baiheng Li, Yi Zheng, Peilong Lai, Jianyu Weng, Donghai Wu, Yuguo Ma, Xudong Chen, Zhesheng Wen, Yaoyu Chen, Xiaoyan Feng, Yangqiu Li, Pentao Liu, Xin Du, Duanqing Pei, Yao Yao, Bing Xu, Ke Ding, Peng Li

**Affiliations:** ^1^ School of Life Science, University of Science and Technology of China, Anhui, China; ^2^ State Key Laboratory of Respiratory Disease, Guangzhou Institutes of Biomedicine and Health, Chinese Academy of Sciences, Guangzhou, China; ^3^ Key Laboratory of Regenerative Biology, South China Institute for Stem Cell Biology and Regenerative Medicine, Guangzhou Institutes of Biomedicine and Health, Chinese Academy of Sciences, Guangzhou, China; ^4^ Guangdong Provincial Key Laboratory of Stem Cell and Regenerative Medicine, South China Institute for Stem Cell Biology and Regenerative Medicine, Guangzhou Institutes of Biomedicine and Health, Chinese Academy of Sciences, Guangzhou, China; ^5^ Department of Hematology, The First Affiliated Hospital of Xiamen University, Xiamen, China; ^6^ Department of Hematology, Guangdong Provincial People's Hospital, Guangzhou, China; ^7^ Yikang Tailai Technology Co. Ltd, Guangzhou, China; ^8^ Department of Interventional Radiology, Shenzhen People's Hospital, Shenzhen, China; ^9^ Department of Thoracic Oncology, Sun Yat-Sen University Cancer Center, Guangzhou, China; ^10^ First Affiliated Hospital of Nanjing Medical University, Jiangsu Province Hospital, Nanjing, China; ^11^ Chongqing HiChuang Biomedical Corp., Chongqing, China; ^12^ Department of Hematology, Medical College, Jinan University, Guangzhou, China; ^13^ Key Laboratory for Regenerative Medicine of Ministry of Education, Jinan University, Guangzhou, China; ^14^ Wellcome Trust Sanger Institute, Hinxton, Cambridge CB10 1HH, England, UK; ^15^ Drug Discovery Pipeline, Guangzhou Institutes of Biomedicine and Health, Chinese Academy of Sciences, Guangzhou, China

**Keywords:** pre-B ALL, GZD824, SRC, PI3K/AKT, PDX

## Abstract

Available therapeutic options for advanced B cell precursor acute lymphoblastic leukemia (pre-B ALL) are limited. Many lead to neutropenia, leaving patients at risk of life-threatening infections and result in bad outcomes. New treatment options are needed to improve overall survival. We previously showed that GZD824, a novel BCR-ABL tyrosine kinase inhibitor, has anti-tumor activity in Philadelphia chromosome-positive (Ph+) chronic myeloid leukemia cells and tumor models. Here, we show that GZD824 decreases cell viability, induces cell-cycle arrest, and causes apoptosis in pre-B ALL cells. Furthermore, Ph– pre-B ALL cells were more sensitive to GZD824 than Ph+ pre-B ALL cells. GZD824 consistently reduced tumor loads in Ph– pre-B ALL xenografts but failed to suppress Ph+ pre-B ALL xenografts. GZD824 decreased phosphorylation of SRC kinase, STAT3, RB and C-myc. It also downregulated the expression of *BCL-XL*, *CCND1* and *CDK4* and upregulated expression of *CCKN1A*. Expression of IRS1 was decreased in GZD824-treated pre-B ALL cells, blocking the PI3K/AKT pathway. These data demonstrate that GZD824 suppresses pre-B ALL cells through inhibition of the SRC kinase and PI3K/AKT pathways and may be a potential therapeutic agent for the management of pre-B ALL.

## INTRODUCTION

Pre-B ALL is characterized by malignant proliferation and accumulation of early B lymphoid precursors in the bone marrow (BM), blood, and lymphoid organs [1] due to acquired mutations in early B cells [2, 3]. Although pre-B ALL is highly responsive to chemotherapy, relapse occurs in approximately 25% of children [4]. The relatively non-specific actions of anti-cancer drugs often result in unacceptable toxicities that can occasionally prove fatal or produce lifelong consequences for survivors [5]. The inability to further intensify current treatments in high-risk patients due to dose-limiting toxicities means that new agents are needed to significantly improve overall survival.

The SRC non-receptor tyrosine kinase is overexpressed and activated in a large number of human malignancies, including ALL [6, 7]. Dysregulation of SRC activity rather than its overexpression is thought to be the key contributor to oncogenesis [8]. Consistent with SRC kinase, the members of the PI3K/AKT pathway are dysregulated in a wide spectrum of human cancers, including leukemia [9, 10]. PI3K inhibitors such as BEZ235 suppress cell proliferation, induce cell death, and extend survival of NOD/SCID mice engrafted with human ALL cells [11]. These findings suggest that inhibition of the SRC kinase or PI3K/AKT pathways in ALL could be an effective new treatment for this disease.

The tyrosine kinase inhibitors (TKI) imatinib (IM), nilotinib, and dasatinib are currently used to treat adult patients with Ph+ ALL and myelodysplastic/myeloproliferative diseases [6]. GZD824, a hybrid molecule of the FDA-approved drugs IM, nilotinib, and dasatinib, has been shown to inhibit the growth of human CML in a xenograft model [12, 13]. The effects of GZD824 on other cancers, including pre-B ALL, have not been evaluated. Here, the efficacy of GZD824, a BCR-ABL TKI, was examined in pre-B ALL cell lines and adult patient primary cells.

## RESULTS

### GZD824 has antitumor activity in human pre-B ALL cells

**Figure 1:**
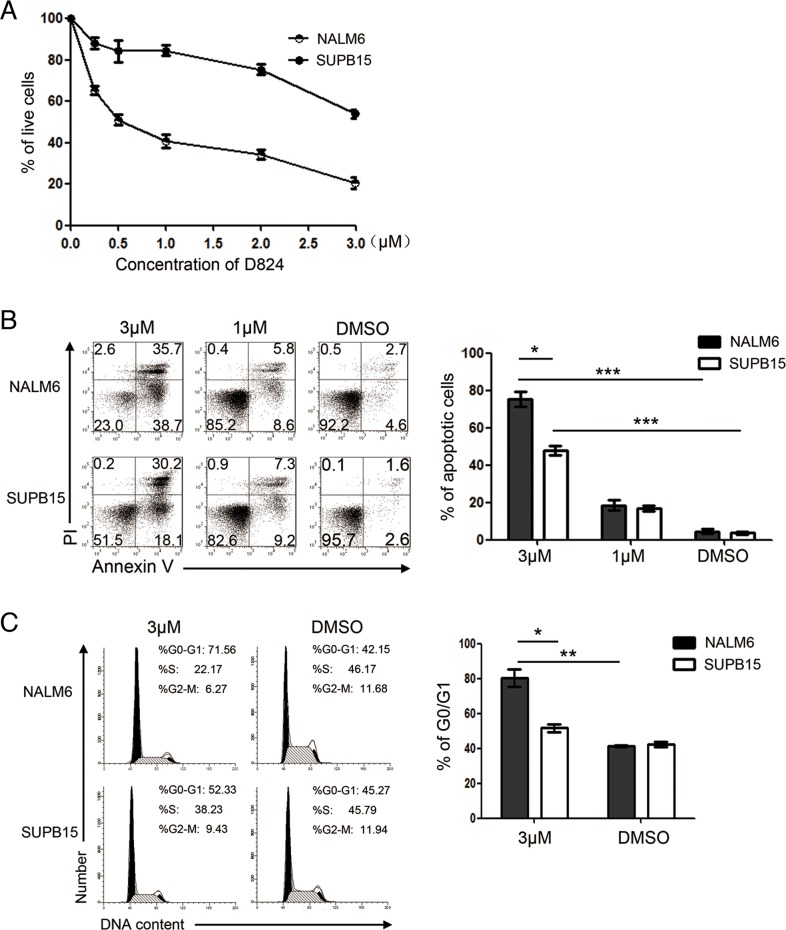
GZD824 inhibits proliferation and induces apoptosis and G0/G1 cell-cycle arrest of human B acute lymphoblast leukemia (pre-B ALL) cell lines **A.** GZD824 cytotoxicity in pre-B ALL cells: NALM6 and SUPB15 were incubated with GZD824 at various concentrations for 24 hours, and viability was determined by CCK8 assay. Viability was calculated relative to time-matched untreated controls. D824 is short for GZD824 in the following figure. **B.** Left: representative flow cytometric analysis of NALM6 and SUPB15 cells treated with DMSO, 1μM, or 3μM of GZD824 for 24 hours. Right: Statistical analysis of AnnexinV-positive cells in GZD824 treated NALM6 and SUPB15 cells. **C.** Left: representative images of cell cycle distribution in NALM6 and SUPB15 cells treated with DMSO or 3μM of GZD824 for 24 hours. Right: Statistical analysis of the distribution of cells in G0/G1 phases in GZD824 treated cells. Data are shown as the mean ± SEM (error bars) from three independent experiments. Significance values: ^*^P<0.05; ^**^P<0.01; ^***^P<0.001.

We previously reported that GZD824, synthesized by hybridizing the structural moieties from the FDA-approved drugs IM, nilotinib, and dasatinib, is capable of inhibiting BCR/ABL kinase and eliminating Ph+ CML cells [13]. We thus assessed the antitumor activity of GZD824 in Ph+ pre-B ALL cells. Surprisingly, we found that GZD824 inhibited the proliferation of the Ph- pre-B ALL cell line (NALM6) with an IC_50_ of 500 nM, much lower than that in the Ph+ pre-B ALL cell line (SUPB15) (Figure 1A). We next investigated whether GZD824 could induce apoptosis in NALM6 or SUPB15 cells. Consistently, 24 h post drug treatment, the apoptotic rates of the NALM6 and SUPB15 cell lines were moderate with GZD824 treatment (1μM). With 3μM GZD824 treatment, 74.4% of the NALM6 cells were undergoing apoptosis (AnnexinV+), compared to only 48.3% of SUPB15 cells (Figure 1B). Application of 500 nM of GZD824 did not increase the percentages of apoptotic NALM6 cells (Supplementary Figure S1A). To test the effects of GZD824 on the cell cycle of pre-B ALL cells, NALM6 and SUPB15 cells were treated with 500nM or 3μM GZD824. 3μM GZD824 treatment increased the proportions of the G_0_/G_1_ fraction in these two cell lines and the percentage of the G_0_/G_1_ fraction in NALM6 was 71.6%, significantly higher than that in SUPB15 (52.2%) (Figure 1C). Moreover, 500 nM GZD824 increased the proportions of the G_0_/G_1_ fraction in NALM6 cells but not in SUPB15 cells (Supplementary Figure S1B). These results demonstrate that GZD824 prevents proliferation and induces apoptosis in both NALM6 and SUPB15 pre-B ALL cell lines. Furthermore, the antitumor activity of GZD824 was more effective for NALM6 than for SUPB15.

**Figure 2:**
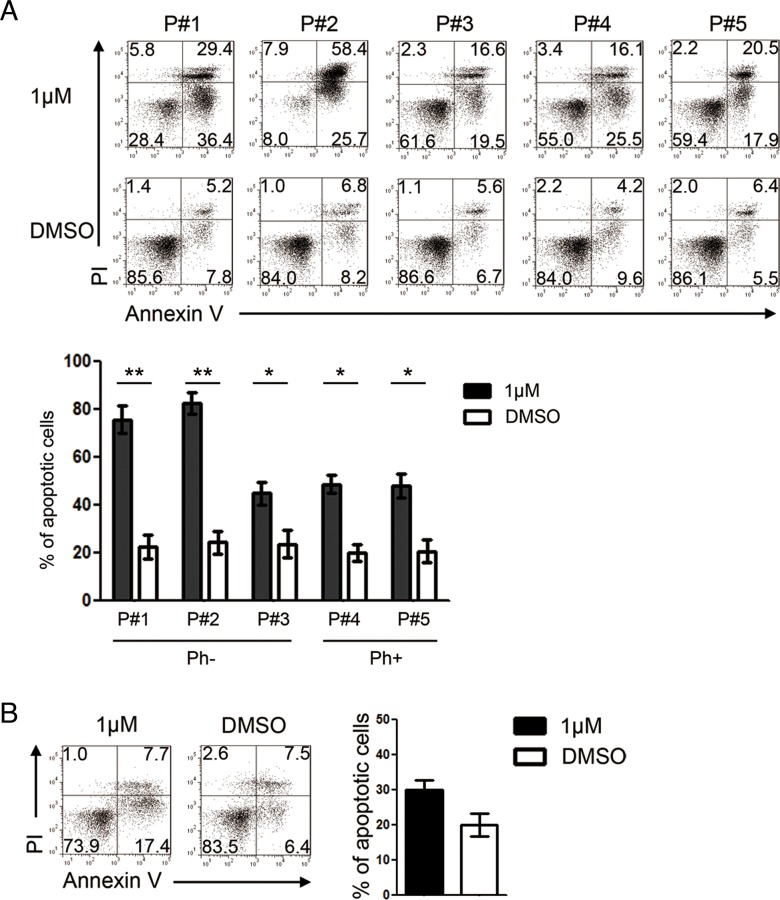
GZD824 induces apoptosis of primary pre-B ALL cells from patients with no toxicity to normal B cells **A.** GZD824 cytotoxicity in primary pre-B ALL cells: primary pre-B ALL cells of P#1, P#2, and P#3 were from Ph- pre-B ALL, and primary pre-B ALL cells of P#4 and P#5 were from Ph+ pre-B ALL. Up: Representative flow cytometric analysis of primary pre-B ALL cells treated with DMSO or 1μM GZD824. Bottom: Statistical analysis of Annexin V-positive cells in GZD824 treated primary pre-B ALL cells. (P#1, P#2, P#3, P#4 and P#5 are short for patient #1, patient #2, patient #3, patient #4, and patient #5) **B.** Left: Representative flow cytometric analysis of normal B cells treated with DMSO or 1μM GZD824. Right: Statistical analysis of AnnexinV-positive cells in GZD824 treated primary B cells. Data are shown as the mean ± SEM (error bars) from three independent experiments. Significance values: ^*^P<0.05; ^**^P<0.01; ^***^P<0.001.

We next characterized the effects of GZD824 on primary pre-B ALL cells from five patients (Supplementary Table S1). Pre-B ALL cells from patient #1 (P#1), patient #2 (P#2), and patient #3 (P#3) were Ph-, whereas those from patient #4 (P#4) and patient #5 (P#5) were Ph+. GZD824 treatment significantly increased the percentages of apoptotic cells in all five samples (Figure 2A). Consistent with the results obtained from NALM6 and SUPB15, the percentages of apoptotic cells in Ph- primary pre-B ALL cells were higher than those in Ph+ primary pre-B ALL cells upon GZD824 treatment (Figure 2A). However, GZD824 treatment did not induce apoptosis in normal human primary B cells (Figure 2B). Taken together, these results show that GZD824 specifically killed primary human pre-B ALL cells *in vitro*, independent of the presence of the Philadelphia chromosome.

### GZD824 inhibits the growth of pre-B ALL cells in patient-derived xenograft (PDX) mouse models

**Figure 3:**
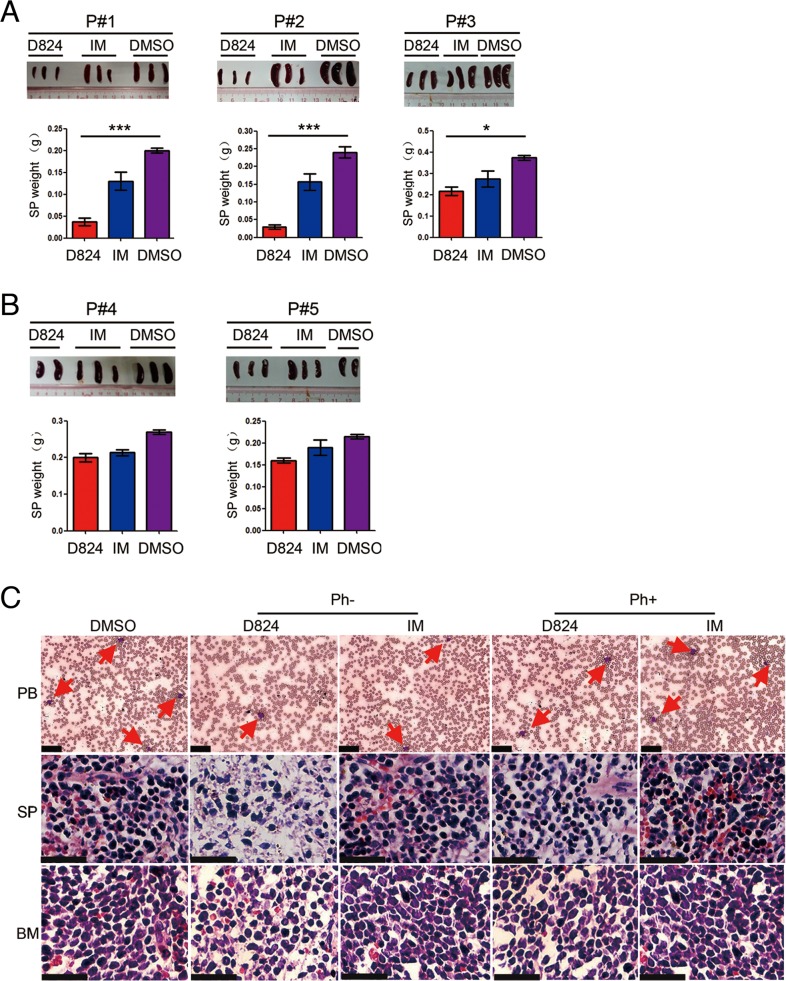
GZD824 inhibits the growth of pre-B ALL cells in PDX models **A.** SP of the mice that were injected with pre-B ALL cells of P#1, P#2, and P#3 with GZD824, imatinib (IM), or DMSO treatment were compared for sizes and weights. Top: The pictures of SP sizes were compared in GZD824, IM, or DMSO group. Bottom: Statistical analysis of SP weight in GZD824, IM or DMSO group **B.** SP of the mice that were injected with pre-B ALL cells of P#4 and P#5 with GZD824, IM, or DMSO treatment were compared for sizes and weights. Top: The pictures of SP sizes were compared in GZD824, IM or DMSO group. Bottom: Statistical analysis of SP weight in GZD824, IM or DMSO group. **C.** PDX mice of P#2 with GZD824 treatment had reduced leukemic infiltration in PB, SP, and BM compared to the mice treated with IM or DMSO. Tissue sections of PB (top), SP (middle), and BM (bottom) were assessed histologically after Wright-Giemsa or H&E staining. Red arrows represent examples of leukemic blasts. Slides were imaged on an Olympus BX46 microscope with an Olympus DP72 camera at ×40 magnifications with 0.5 apertures; Olympus cellSens Standard 1.5 image acquisition software was used. Scale bar = 25 μm in all photomicrographs. Data are shown as the mean ± SEM (error bars) from three independent experiments. Significance values: ^*^P<0.05; ^**^P<0.01; ^***^P<0.001.

To evaluate the anti-pre-B ALL activity of GZD824 *in vivo*, we generated human pre-B ALL PDX models by transplanting splenic pre-B ALL cells from the five patient-derived (Supplementary Table S1) xenograft mouse lines into NOD-*scid-IL2Rg−/−* (NSI) mice [18–21]. GZD824, IM, and DMSO treatment were started when the pre-B ALL cells in the PB of the xenograft reached 1%±0.2% of the total (Supplementary Figure S2A and Supplementary Figure S2C). IM was used as a positive control because it is a TKI used in the treatment of multiple cancers, most notably Ph+ CML [6, 7]. We culled the mice after 2 weeks of drug treatment and found that the weights of the SP in the mice that were injected with pre-B ALL cells of P#1, P#2, and P#3 from the GZD824 group were significantly lighter than those from either the IM group or DMSO group (Figure 3A). However, there were no significant differences in SP weights among these three groups of mice with reconstitution of pre-B ALL from patients P#4 and P#5 (Figure 3B). Consistently, PDX mice with Ph- cells that received GZD824 treatment showed reduced leukemic burden in SP and BM compared with those treated with IM or DMSO (Figure 3C).

**Figure 4:**
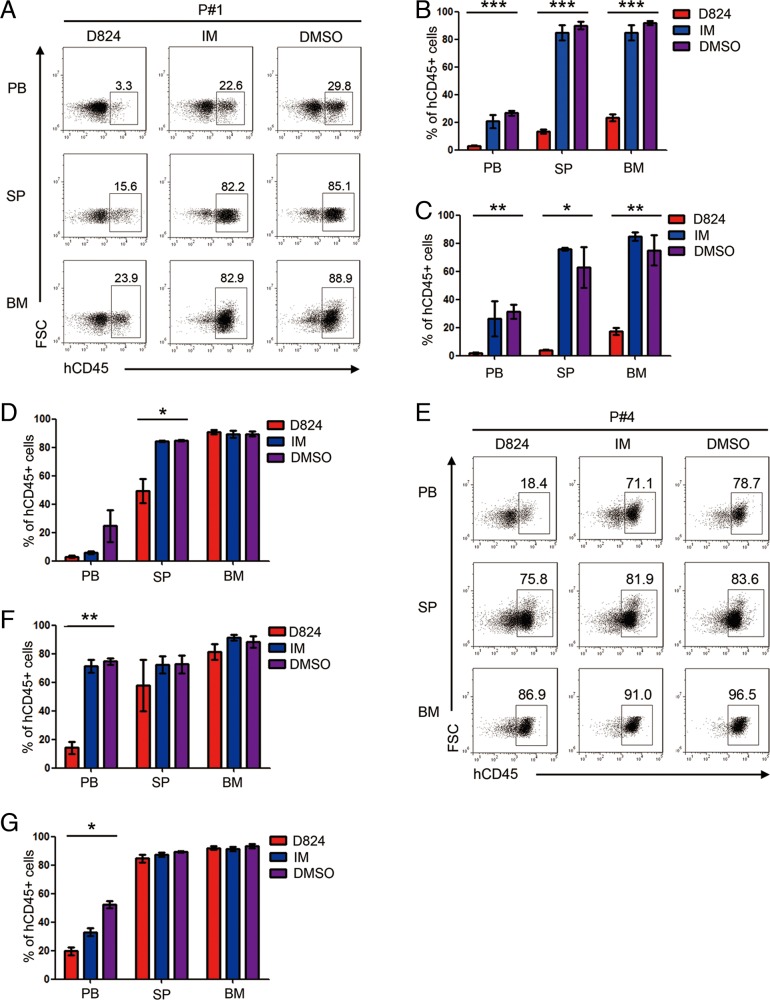
Effects of GZD824 on pre-B ALL cells engraftment **A.** Representative flow cytometric analysis of human CD45+ cells in PB (top), SP (middle), and BM (bottom) of P#1-engrafted mice after treatment with GZD824, IM, or DMSO. **B-D.** The percentages of human CD45+ cells in the PB, SP, and BM of mice transplanted with pre-B ALL cells of P#1 (B), P#2 (C), and P#3 (D) treated under different conditions were measured when the mice were culled. **E.** Representative flow cytometric analysis of human CD45+ cells in PB (top), SP (middle), and BM (bottom) of P#4-engrafted mice after treatment with GZD824, IM, or DMSO. **F-G.** The percentages of human CD45+ cells in the PB, SP, and BM of mice transplanted with pre-B ALL cells of P#4 (F) or P#5 (G) treated under different conditions were measured when the mice were culled. Significance values: ^*^P<0.05; ^**^P<0.01; ^***^P<0.001.

We subsequently analyzed the residual pre-B ALL cells in the PDX mice 2 weeks after treatment with GZD824, IM, or DMSO and found that the PDX mice with Ph- pre-B ALL cells (P#1, P#2, and P#3) that were treated with GZD824 had significantly lower percentages of pre-B ALL cells in PB, SP, and BM than those from the IM or DMSO groups (Figure 4A–4D), despite the observation that the reduction of tumor load in the BM of xenografts from P#3 is not significant in the GZD824 group compared to IM and DMSO groups. In contrast, GZD824 treatment reduced the number of circulating pre-B ALL cells in the PB of the Ph+ pre-B ALL PDX mice but failed to reduce the residual pre-B ALL cells in SP or BM (Figure 4E–4G). IM did not significantly inhibit the growth of either Ph+ or Ph- pre-B ALL cells in the PDX models (Figure 4A–4G). Taken together, these results show that GZD824 more effectively suppresses the growth of human Ph- pre-B ALL cells than that of Ph+ pre-B ALL cells *in vivo*.

### GZD824 inhibits the SRC kinase in pre-B ALL cells

**Figure 5:**
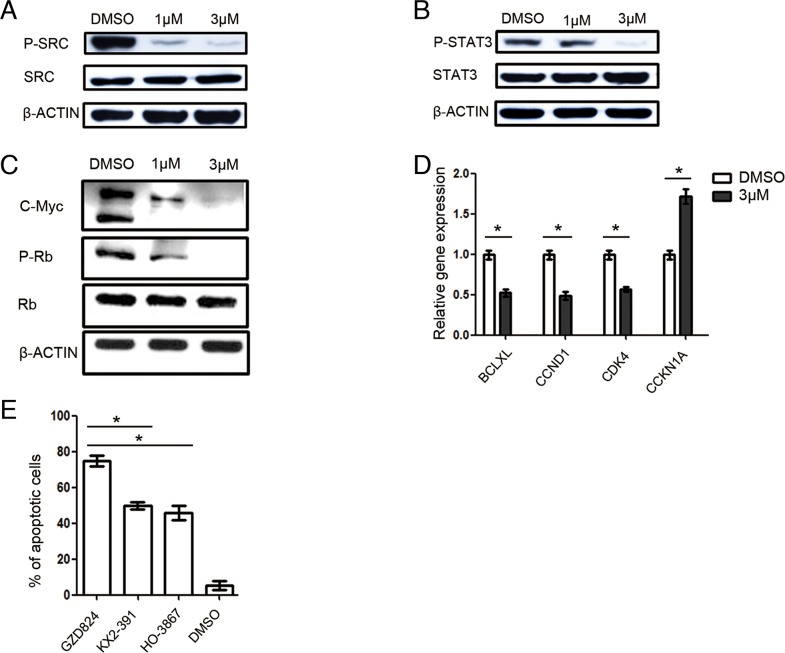
GZD824 inhibits the SRC kinase in pre-B ALL cells **A-C.** Western blot analysis of D824-treated NALM6 cells. Cells were treated with DMSO, GZD824 (1μM), or GZD824 (3μM) for 24 hours. Lysates were analyzed with antibodies to (A) phospho-SRC and SRC; (B) phospho-STAT3 and STAT3; and (C) phospho-Rb, Rb, and C-Myc. **D.** qRT-PCR products of *BCL-XL*, *CCND1, CDK4*, and *CCKN1A* mRNA were normalized to GAPDH and presented as fold increase or decrease compared to control, 24 hours after treatment with 3 μM GZD824. **E.** NALM6 cells were treated with GZD824, SRC inhibitor (KX2-391), STAT3 inhibitor (HO-3867), or DMSO for 24 hours. Statistical analysis of Annexin V-positive cells in 3μM of drug treated NALM6 cells. All data from independent experiments are presented as mean ± SEM. Significance values: ^*^P<0.05; ^**^P<0.01; ^***^P<0.001.

To elucidate the mechanism of GZD824 inhibition for pre-B ALL cells, kinase assays were performed to determine possible binding targets of GZD824. Besides ABL kinases and phosphorylated ABL kinases, GZD824 bound to the SRC kinase with high affinity (Supplementary Table S2). The inhibition of the SRC/STAT3 signal pathways causes cell-cycle arrest and apoptosis [22]. We thus determined that GZD824 treatment induces pre-B ALL cells to undergo apoptosis through the SRC/STAT3 pathway. We evaluated the effects of GZD824 on the SRC/STAT3 pathway in NALM6 cells by measuring the phosphorylation profiles of SRC and STAT3. As expected, GZD824 treatment reduced both phosphorylated SRC (Figure 5A) and phosphorylated STAT3 in NALM6 cells (Figure 5B). We also found that GZD824 downregulated c-Myc oncogene expression and suppressed Rb phosphorylation (Figure 5C). Further evidence of GZD824 inhibition of SRC/STAT3 activity was derived by gene expression analysis of several STAT3 target genes. Treatment with GZD824 caused further downregulation of genes known to be positively regulated by STAT3, such as the anti-apoptotic gene *BCL-XL* [23], the positive cell cycle regulators *CCND1*[24] and *CDK4* [25] and the negative cell cycle regulator *CDKN1A* [24] (Figure 5D).

To test whether STAT3 is the main target of the SRC signaling pathway in pre-B ALL, we treated NALM6 cells with GZD824, SRC inhibitor (KX2-391), STAT3 inhibitor (HO-3867), or DMSO. We found that KX2-391 and HO-3867 induce similar amounts of apoptosis, demonstrating that STAT3 is the main target of the SRC signaling pathway in pre-B ALL (Figure 5E). In addition, GZD824 showed a significantly increased apoptotic level compared to KX2-391, HO-3867 and DMSO (Figure 5E). Accordingly, in addition to the SRC/STAT3 signaling pathway, other signaling pathways may be targeted by GZD824 inhibition.

### IGF-1 rescues GZD824 inhibition via the IRS1/PI3K/AKT signaling pathway

**Figure 6:**
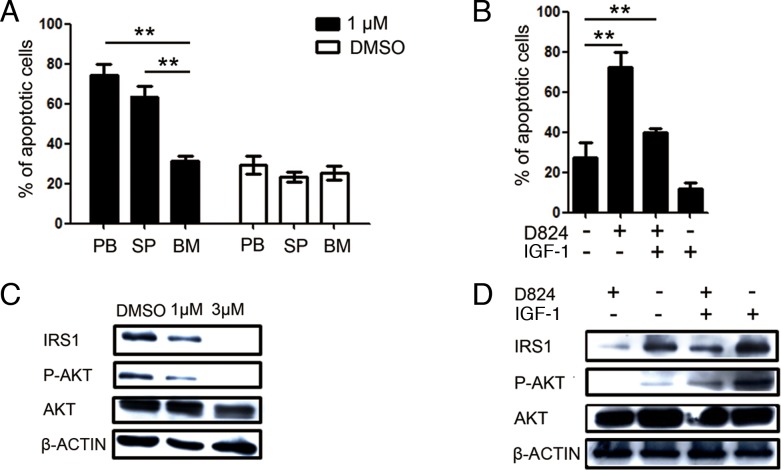
GZD824 inhibits PI3K/AKT signaling in pre-B ALL cells **A.** Cells were harvested from PB, SP, and BM of P#2 xenograft mice and cultured with or without 1μM GZD824 for 24h. Bars represent the mean percentages of AnnexinV+ cells in each group. **B.** Primary pre-B ALL cells from spleen of P#1 xenograft mouse treated with DMSO, GZD824 (1μM), IGF-1 (10ng/ML), or both. Bars represent the mean percentages of AnnexinV+ cells in each group. **C.** Western blot analysis of NALM6 cells. Cells were treated with DMSO, GZD824 (1μM), or GZD824 (3μM) for 24 hours. Lysates were analyzed with antibodies against IRS1, phosphate AKT, and AKT. **D.** Western blot analysis of primary pre-B ALL cells. Primary cells treated with GZD824 (1μM), IGF-1 (10ng/ML), or both for 24 hours. Lysates were analyzed with antibodies against Irs1, phosphate AKT, and AKT.

The percentages of residual pre-B ALL cells in PB, SP, and BM were different in individual PDX mice upon GZD824 treatment (Figure 4A–4G), suggesting that pre-B ALL cells in these compartments showed different resistance capacities to GZD824. Therefore, we compared the GZD824 sensitivity among the pre-B ALL cells harvested from the PB, SP, and BM of the same pre-B ALL PDX mice. Before *in vitro* assay, the pre-B ALL cells from different organs were purified with human anti-CD45+ by MACS (Supplementary Figure S3A-B). Interestingly, compared to the pre-B ALL cells derived from PB and SP, the percentages of apoptotic pre-B ALL cells derived from BM were significantly lower than those from SP and PB 24 hours after GZD824 treatment *in vitro* (Figure 6A), suggesting that the pre-B ALL cells from BM were more tolerant to GZD824 treatment. We previously found that insulin-like growth factor 1 (IGF-1), which is highly expressed in BM, increased the resistance capacity of pre-B ALL cells to chemotherapy via the PI3K/AKT pathway (Zhiwu Jang unpublished). Thus, we hypothesized that IGF-1 would rescue pre-B ALL cells from GZD824 inhibition by up-regulating the PI3K/AKT pathway. Primary pre-B ALL cells treated with GZD824 alone showed much higher apoptotic levels than those cells treated with both GZD824 and IGF-1 (Figure 6B), suggesting that IGF-1 decreased the sensitivity of pre-B ALL cells to GZD824 treatment.

**Figure 7:**
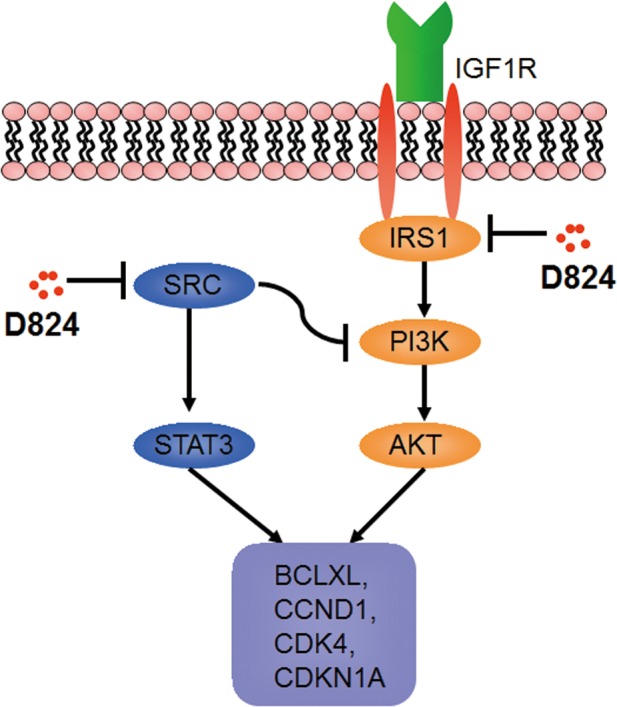
A proposed mechanism for the anticancer activity of GZD824 A detailed description of the mechanism can be found in the results and discussion sections.

PI3K/AKT signaling is the major pathway controlled by IGF-1[26]. Binding of IGF-1 with IGF-1R induces tyrosine phosphorylation of insulin receptor substrate 1 (IRS1), which in turn activates PI3K and its downstream target, AKT [27, 28]. Our observation that IGF-1 rescued the inhibition of pre-B ALL cells by GZD824 treatment led us to hypothesize that GZD824 would directly restrict IRS1 expression, which would result in a subsequent activation of AKT in pre-B ALL cells. To test this hypothesis, NALM6 cells were treated with GZD824, followed by analysis of the levels of proteins related to IGF-1 signaling and AKT phosphorylation. The levels of IRS1 protein and phosphorylated AKT (Ser473) were reduced in the GZD824-treated NALM6 cells (Figure 6C). Furthermore, the primary pre-B ALL cells with lower expression of IRS1 (Figure 4A–4G) were more sensitive to GZD824 treatment in xenografts (Supplementary Figure S4). Consistently, primary pre-B ALL cells treated with GZD824 resulted in a reduction in IRS1 and phosphorylated AKT (Ser473) (Figure 6D). As expected, IGF-1 significantly increased the levels of IRS1 and phosphorylated AKT (Ser473) in primary pre-B ALL cells and reversed increases in IRS1 and phosphorylated AKT in primary pre-B ALL cells after treatment with GZD824 (Figure 6D). Collectively, these studies suggest that GZD824 negatively regulated the survival and proliferation of pre-B ALL cells by inhibiting the SRC kinase and PI3K/AKT signaling pathways (Figure 7).

## DISCUSSION

We previously showed that GZD824 suppresses Ph+ CML cells and induces tumor regression in mouse xenograft tumor models by inhibiting BCR-ABL kinase [16, 17]. Here we found that GZD824 treatment significantly decreased proliferation of both human Ph+ and Ph– pre-B ALL cells, indicating that GZD824's anti-tumor activity in pre-B ALL is partly independent of BCR-ABL kinase activity. Abnormal interactions between BCR-ABL leads to activation of BCR-ABL tyrosine kinase, SRC kinases, the PI3K pathway, Ras-mitogen-activated protein kinase (Ras/MAPK), and the Janus-activated kinase (JAK) pathway [29, 30]. SRC kinases remain active after IM treatment in Ph+ leukemia [31]. Although GZD824 does not bind to JAK or MAPK, as revealed in the present kinase assay results, MAPK or JAK might still be activated in GZD824-treated pre-B ALL cells. This may explain the relatively poor activity of IM and GZD824 against Ph+ pre-B ALL. Moreover, it is therefore possible to efficiently eradicate Ph+ pre-B ALL cells by combining multiple TKIs that target these pathways.

Ph– pre-B ALL cells were more sensitive to GZD824 than Ph+ pre-B ALL cells both *in vitro* and *in vivo*, suggesting that other mechanisms are involved in the action of GZD824 inhibition of pre-B ALL. Levels of both P-SRC and P-STAT3, a downstream phosphorylation target of SRC, were significantly reduced after treatment with GZD824 in Ph– pre-B ALL cells (Figure 7). GZD824 suppressed both SRC and PI3K/AKT in Ph– cells, which is consistent with other reports that inhibition of SRC kinase or PI3K/AKT signaling suppresses ALL cells *in vitro* and *in vivo* [11, 32–34]. To our knowledge, GZD824 is the first compound to show an antitumor effect in pre-B ALL by inhibiting both the SRC kinase and IRS1/PI3K/AKT pathways (Figure 7). IRS1 is an important mediator between IGF-1 receptors and the PI3K/AKT pathway [35, 36]. An earlier study suggested that IRS1 expression correlates negatively with survival in patients with Ph+ ALL [37]. In the present study, levels of IRS1 expression were negatively correlated with GZD824 treatment outcomes in xenografts (Supplementary Figure S4). Thus IRS1 could potentially be used as a biomarker to predict GZD824 efficiency in treating pre-B ALL in the future.

In sum, our findings indicate GZD824 is cytotoxic to pre-B ALL cell lines and primary cells *in vitro* and in xenografts. Moreover, GZD824 specifically inhibits SRC kinase and PI3K/AKT, making it a highly promising agent for clinical testing for the treatment of acute pre-B ALL.

## MATERIALS AND METHODS

### GZD824 compound and other chemicals

GZD824 was synthesized in Ke Ding's laboratory. Compound solutions were freshly prepared by dissolving the compounds in dimethyl sulfoxide (DMSO) (D2650, Sigma Aldrich, St. Louis, MO, USA) before each experiment. IM (SML1027, Sigma Aldrich, St. Louis, MO, USA) was purchased from Sigma Aldrich.

### Cell culture

Human pre-B ALL cell lines NALM6 and SUPB15 were purchased from American Type Culture Collection (ATCC, Maryland, USA). All pre-B ALL cell lines were maintained in cell culture media and conditions as recommended by the ATCC. Cells were treated with GZD824 for 24 hours. GZD824 (3μM, 1μM, or 0.5μM) was used in the experiments unless otherwise noted. NALM6 and SUPB15 cells were maintained in RPMI-1640 medium (Gibco, New York, NY, USA) with 10% fetal bovine serum (Biochrom, Melbourne, Australia).

Human pre-B ALL cells were obtained from spleen (SP) of sick pre-B ALL xenograft mice. Human CD45+ pre-B ALL cells, accounting for over 95% of the total SP mononuclear cells, were subjected *in vitro* assay. To determine the *in vitro* anticancer efficacy of GZD824, the drug was applied to fresh splenic pre-B ALL cells that were directly isolated from sick pre-B ALL xenograft mice in 3 independent experiments. In addition, fresh splenic cells were analyzed 24 hours post drug treatment. At this time point about 20% of the total cells in the vehicle group are apoptotic. We did not add any cytokine cocktails during the drug efficacy test. All cells were cultured at 37°C in 5% carbon dioxide and a normal level of oxygen.

### Isolation of human pre-B ALL cells

All pre-B ALL samples were obtained from the Nanfang Hospital, Southern Medical University. For all of the patients who participated in this study, written informed consent was obtained, which was approved by the Ethics Committee of Guangzhou Institutes of Biomedicine and Health, Chinese Academy of Sciences. Human pre-B ALL cells were isolated with Lymphoprep (StemCell Technologies, Canada) according to the manufacturer's instructions.

### Proliferation assays

Cells were seeded at a density of 0.5×10^6^ to 1×10^6^ cells per ml in the presence or absence of inhibitor for the indicated times. Cell proliferation was measured 24 hours after treatment, using the Cell Counting Kit-8 assay (CCK-8, DOJINDO, Kumamoto, Japan). The proliferation analysis of each group in each individual experiment and throughout the whole experimental process was performed in triplicate.

### Cell-cycle analysis and detection of apoptotic cells

Cell cycle analysis was performed by flow cytometry after propidium iodide (PI) staining as described [14, 15]. Approximately 2 × 10^6^ cells were suspended in 0.5 ml of phosphate-buffered saline (PBS) and vortexed with 3 ml of 80% ethanol. Cells were fixed at 4°C for 1 hr, washed with PBS, re-suspended in PBS, and vortexed vigorously. Cells were re-suspended in 1 ml of PBS containing 50 μg/ml of DNase-free RNase A (EN0531, Thermo Fisher scientific, Waltham, USA) and 50 μg/ml of propidium iodide PI (P4170, Sigma Aldrich, St. Louis, MO, USA). Cells were stained for at least 1 hr at room temperature in the dark. Stained cells were then subjected to flow cytometric analysis by an Accuri™ C6 (BD Biosciences, CA, USA).

For identification of apoptotic cells among NALM6, SUPB15, and primary pre-B ALL cells, the GZD824-treated cells were stained with Annexin V Apoptosis Detection Kit (88-8007, BD ebioscience, CA, USA) according to the manufacturer's instructions (Affymetrix ebioscience, CA, USA) and then subjected to flow cytometric analysis by the Accuri™ C6 (BD Biosciences, CA, USA). For the *in vitro* drug sensitivity test in Figure 6A, the human CD45+ cells in spleen and bone marrow from sick mice account for over 95% of the total. We enriched human CD45+ cells in PB by MACS according to the manufacturer's instruction. After the enrichment, the purity of human CD45+ cell in PB reached 95%. The percentage of apoptotic cells was demarcated as the amount of both early and late facets of apoptosis (annexin V positive), as previously described [16]. All data were analyzed with FlowJo software (Tree Star, Inc., Ashland, USA).

### H&E staining

Peripheral blood (PB), spleen (SP), and bone marrow (BM) from mice sacrificed by CO_2_ asphyxiation were dissected and paraffin embedded for histological staining. All protocols used in this study were approved by the animal protocol committee of the Guangzhou Institutes of Biomedicine and Health (GIBH).

### Active-site-dependent competition kinase binding assay

Kinase assay was performed as previously described [12, 17]. The binding activities of GZD824 were analyzed by KINOME scan system conducted by Ambit Bioscience (CA, USA). Briefly, kinases were tagged with DNA. The ligands were biotinylated and immobilized to streptavidin coated beads. The binding reactions were assembled by incubating DNA-tagged kinases, immobilized ligands, and test compounds in binding reactions (20% SeaBlock, 0.17×PBS, 0.05% tween-20, 6 mM DTT) for 1.0 h at room temperature. The affinity beads were washed with washing buffer (1×PBS, 0.05% Tween-20) first and then elution buffer (1×PBS, 0.05% Tween 20, 0.5μM nonbiotinylated affinity ligands). The kinase concentration in the eluate was determined by quantitative PCR of the DNA tagged to the kinase. The ability of the test compound to bind to the kinase was evaluated with percent control (%) as (test compound signal −positive control signal)/negative control signal−positive control signal) ×100%. Negative control is DMSO control (100% ctrl) and positive control is control compound (0% ctrl).

### Western blot analysis

For Western blot analysis, 30 μg of total protein extracts prepared from NALM6 or primary pre-B ALL cells from SP of PDX mice were separated on SDS-PAGE and transferred to a PVDF membrane (Bio-Rad, Hercules, CA, USA). The following commercially available antibodies and dilutions were used for Western blotting: human anti-STAT3 (ab119352, Abcam, UK), human anti-phospho-STAT3 (Y705) (ab76315, Abcam, UK), human anti-SRC (ab32102, Abcam, UK), human anti-phospho-SRC (Y419) (ab185617, Abcam, UK), human anti-RB (9313, Cell Signaling Technology, MA, USA), human anti-phospho–RB (Ser807/811) (8516, Cell Signaling Technology, MA, USA), human anti-C-myc (13987, Cell Signaling Technology, MA, USA), anti-AKT (pan) (4691P, Cell Signaling Technology, MA, USA), anti-phospho-AKT (Ser473) (4060P, Cell Signaling Technology, MA, USA), anti-IRS-1 (2390S, Cell Signaling Technology, MA, USA), and human anti–β-actin (4970, Cell Signaling Technology, MA, USA). Western blot analysis was performed with cell lines or primary pre-B ALL cells from SP following GZD824 treatment, and signal intensities were normalized to β-actin signals.

### Transplantation experiments

Animal experiments were performed in the Laboratory Animal Center of GIBH, and all animal procedures were approved by the Animal Welfare Committee of GIBH. All protocols were approved by the relevant institutional animal care and use committee (IACUC). Bone marrow samples were acquired at diagnosis from pre-B ALL patients. Leukemia blasts were enriched by Ficoll-Paque centrifugation and were washed, precipitated, and pelleted in PBS. Then, Leukemia blasts were enriched for human CD45+ cells by MACS according to the manufacture instruction. Freshly isolated primary CD45+ cells were transplanted into NODSCID-IL2RG−/− (NSI) mice to establish P1 PDX mice. P1 PDX mice were established by intravenously engrafting 2×10^6^ CD45+ cells into 8-week-old, sub-lethally irradiated NSI mice. P2 to Pn (n<5) PDX mice were established by intravenously injecting 1×10^6^ splenic pre-B ALL cells from sick pre-B ALL xenograft mice into 8-week-old, sub-lethally irradiated NSI mice. The hCD45+ pre-B ALL cells in the sick PDX mouse, which account for over 95% of the total, were used for further transplantation. Freshly isolated splenic pre-B ALL cells were transplanted into NSI mice. Cell viability, as determined by trypan blue exclusion staining, was typically >90%. If the viability of cells was less than 80%, dead cells were eliminated using a “dead cell removal kit” (Miltenyi Biotec Inc., Germany).

Compound treatment started when the percentage of pre-B ALL cells in PB of xenograft mice reached 1 ± 0.2%. To remove the red blood cells, the PB and SP were treated with 1 × RBC Lysis Buffer (eBioscience) according to the manufacturer's instructions. The peripheral blood mononuclear cells (PBMCs) were analyzed by human anti-CD45 antibody and analyzed with an Accuri C6 (BD bioscience). Mice injected with tumor cells were randomly divided into three groups: the control group, treated with DMSO; the IM group, treated with IM (50 mg/kg body weight) dissolved in PBS; and the GZD824 treatment group, treated with GZD824 (25 mg/kg body weight) dissolved in DMSO solution. Mice received DMSO, IM (50 mg/kg), or GZD824 (25 mg/kg) by intragastric administration daily for two weeks.

### Flow cytometry analysis

Cells were isolated from PB, BM, and SP for flow cytometric analyses. For analysis, the human pre-B ALL cells were labeled with anti-hCD45-PE. The antibody was obtained from eBioscience (San Diego, CA, USA) unless specifically stated. Flow cytometric analysis was performed using an Accuri™ C6 or FACSAria™ II (BD Biosciences, CA, USA). All data were analyzed with FlowJo software (Tree Star, Inc., Ashland, USA).

### qRT-PCR

RNA was extracted using TRIzol reagent (15596-026, Invitrogen, Grand Island, NY, USA), according to the manufacturer's recommended protocol. qRT-PCR was performed using Applied Biosystems StepOne and StepOne Plus Real-Time PCR Systems (Foster City, CA, USA). The experiments were repeated a minimum of three times to confirm the results.

### Statistical analyses

Data were analyzed with the Student *t*-test using GraphPad Prism 5 software. P values of less than 0.05 were considered statistically significant.

## SUPPLEMENTARY FIGURES AND TABLES


