# Supplementary material for: GZD824 suppresses the growth of human B cell precursor acute lymphoblastic leukemia cells by inhibiting the SRC kinase and PI3K/AKT pathways
Source: Oncotarget. 2016 Jul 28;8(50):87002–15. doi: 10.18632/oncotarget.10881 (PMC5675611; doi:10.18632/oncotarget.10881)
Supplement: Supplementary file 1 [file oncotarget-08-87002-s001.pdf]

GZD824 suppresses the growth of human B cell precursor acute lymphoblastic leukemia cells by inhibiting the SRC kinase and PI3K/AKT pathways

SUPPLEMENTARY FIGURES AND TABLES

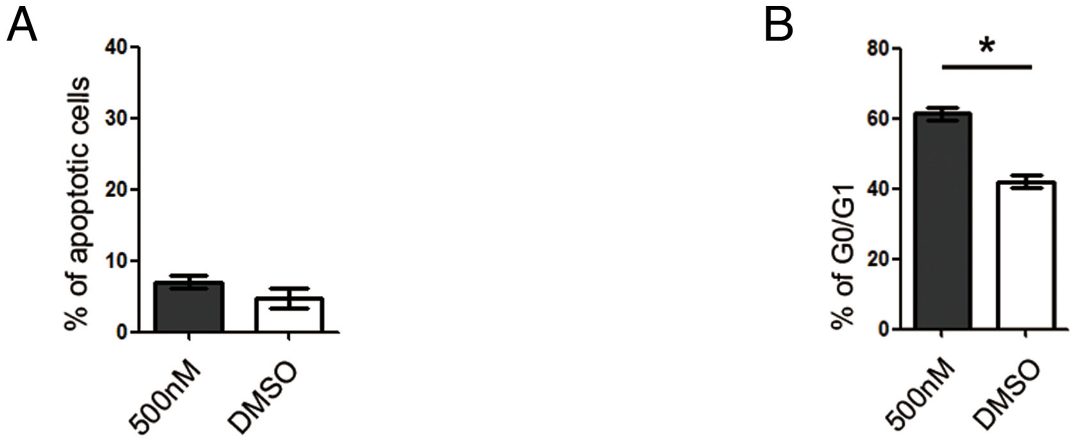

Supplementary Figure S1: Application of 500 nM of GZD824 inhibited cell cycle but did not increase the percentages of apoptotic NALM6 cells. A. Statistical analysis of AnnexinV-positive cells in 500 nM of GZD824 or DMSO treated NALM6 cells. **B.** Statistical analysis of the distribution percentage of cells in G0/G1 phases in 500 nM of GZD824 or DMSO treated NALM6 cells. Data are shown as the mean ± SEM (error bars) from three independent experiments. Significance values: $^{*}P<0.05$; $^{**}P<0.01$; $^{***}P<0.001$.

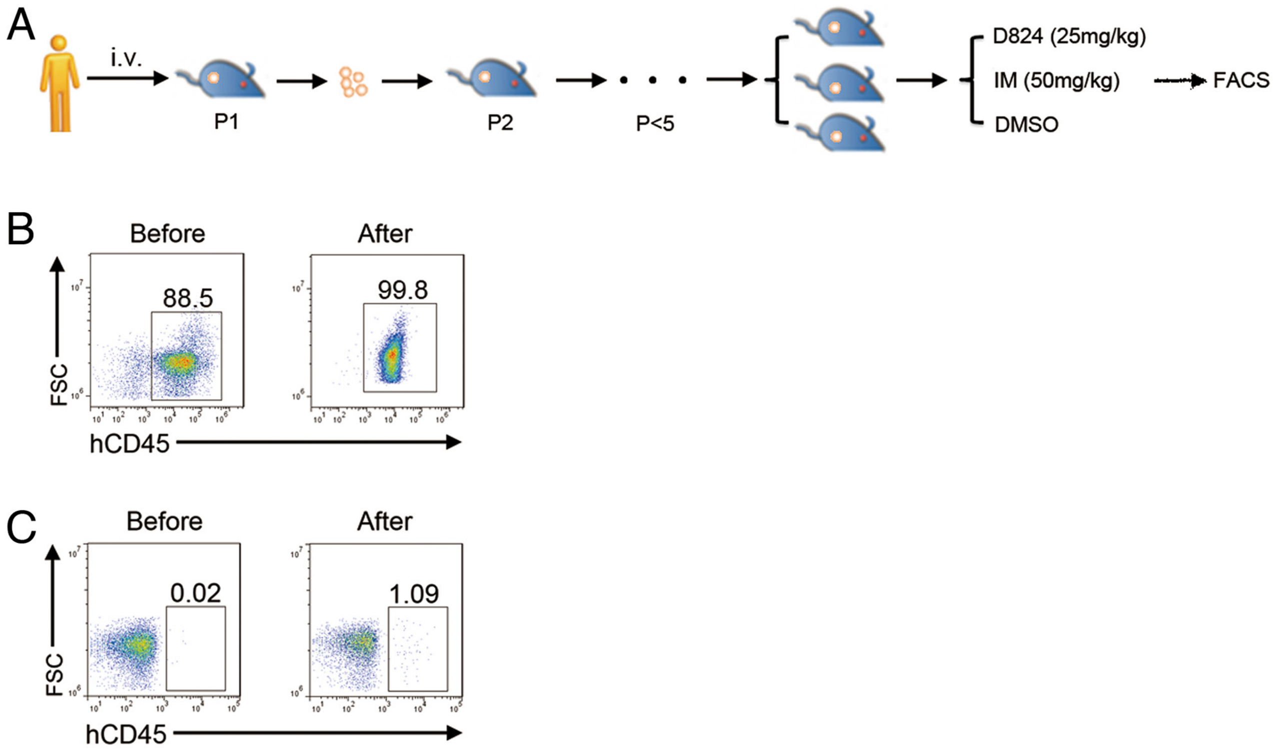

Supplementary Figure S2: Experimental design of the in vivo experiments. A. Experimental design of the in vivo experiments. Splenic pre-B ALL cells (1×10^6) from 5 patient PDX mice were injected intravenously into 8-week-old, sublethally irradiated NSI mice (3 mice/per group). Treatment began when the concentration of human CD45+ in the peripheral blood (PB) of xenograft mice reached 1%±0.2%. D824 (25 mg/kg), IM (50 mg/kg), and DMSO were administered daily over 2 weeks. **B.** Representative FACS dot plots show the percentage of human CD45+ cells in human BM sample before and after sorting by MACS. **C.** Representative FACS dot plots show the percentage of human CD45+ cells in xenograft murine PB before and after transplantation.

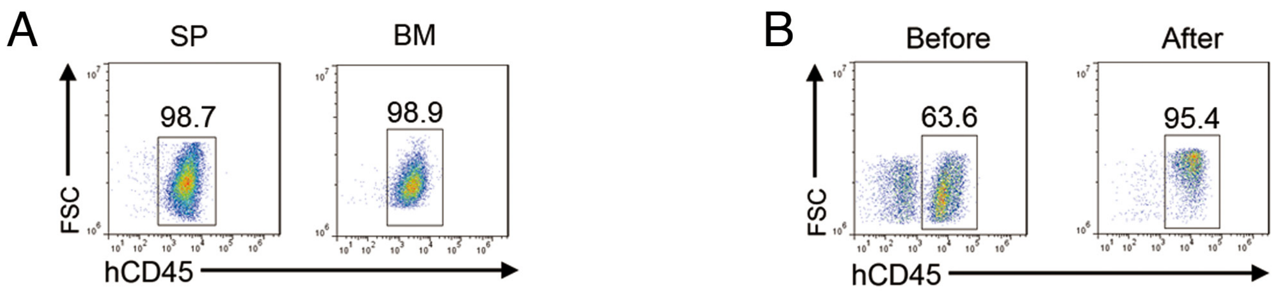

Supplementary Figure S3: Enrichment of human pre-B ALL cells in the organs of sick PDX mice for apoptosis assay. A. Representative FACS dot plots show the percentage of human CD45+ cells in xenograft murine SP and BM **B.** Representative FACS dot plots show the percentage of human CD45+ cells in xenograft murine PB before and after sorting by MACS.

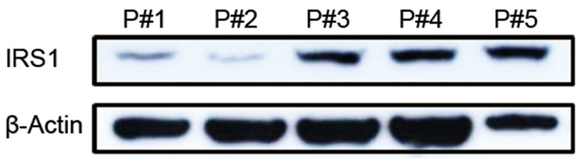

Supplementary Figure S4: Western blot analysis of primary B-ALL cells. Primary cells from P#1, P#2, P#3, P#4, and P#5 were lysed and analyzed with antibody to the IRS1.

Supplementary Table S1: B-ALL patient characteristics

Patient	Age	Sex	ALL lineage	Disease at biopsy	cytogenetic	Immune-phenotype	Current stage
1	21	M	Ph− common ALL	diagnosis	Normal cytogenetics	CD34,CD33,CD19,CD13,CD9, HLA-DR	Poor remission
2	19	F	Ph− common ALL	relapse	Normal cytogenetics	CD10,CD19,CD22, HLA-DR	Poor remission
3	20	M	Ph− common ALL	diagnosis	Normal cytogenetics	CD34,CD9,CD10,CD19,CD22, CD56,HLA-DR	Poor remission
4	58	M	Ph+ common ALL	diagnosis	Normal cytogenetics	CD79a,CD34,CD9,CD10, CD19,CD22,HLA-DR	Poor remission
5	42	M	Ph+ common ALL	diagnosis	46,XY,t(9;22)(q34;q11)	CD34,CD9,CD10,CD19, CD20,HLA-DR	Good remission

Supplementary Table S2: Binding Affinity of GZD824 to multi-kinases as determined by an Active-Site-Dependent Competitive Binding Assay (KINOMEscan Screening) a

Kinase Target	D824	
	%Ctrl@10nM	%Ctrl@100nM
ABL	2.8	0.15
SRC	26	0.75
JAK	100	100
MAPK	81	81

The data represent the mean values of two independent experiments
